# *Staphylococcus aureus* bacteremia at a referral medical center in Kenya: A retrospective review of cases from 2010 to 2018

**DOI:** 10.1371/journal.pone.0234914

**Published:** 2020-06-23

**Authors:** Jennifer M. Fernandez, Jenna B. Dobrick, Afraaz Jadavji, Rodney D. Adam

**Affiliations:** 1 University of Arizona College of Medicine, Tucson, AZ, United States of America; 2 University of Waterloo, Waterloo, ON, Canada; 3 Aga Khan University, Nairobi, Kenya; George Washington University, UNITED STATES

## Abstract

**Background:**

Many studies have shown that *Staphylococcus aureus* is a leading cause of both community onset and hospital onset bloodstream infections. However, relatively little is known about the occurrence and outcome of *S. aureus* bacteremia in sub-Saharan Africa. A previous report indicated that *S. aureus* accounts for 16% of community onset and 6% of hospital onset bloodstream infections at Aga Khan University Hospital Nairobi (AKUHN). Data about the etiology of *S. aureus* bacteremia in sub-Saharan Africa will help optimize recognition and treatment. This study was performed in order to understand the etiologies and risk factors for *S*. *aureus* bacteremia in a sub-Saharan location.

**Materials and methods:**

A review of the electronic record of laboratory results from September 2010 through December 2018 identified 201 patients seen at AKUHN with *S. aureus* bacteremia. The source and/or focus of infection was identified and in-hospital mortality was determined. Cases with bacteremia after three days of hospitalization were considered hospital acquired. Community onset cases were divided into community acquired and health care associated.

**Results:**

Most cases (71%; 143/201) were community onset, but only 41% (83/201) of these cases were community acquired. The most commonly identified foci of infection for community acquired bacteremia were musculoskeletal (25%; 21/83) and skin and soft tissue (24%; 20/83). The majority of health care associated (70%; 40/57) and hospital acquired cases (74%; 43/58) were associated with invasive vascular devices, with peripheral IVs being the most common for hospital acquired and dialysis catheters being the most common for health care associated infections. In-hospital mortality rates were 23% (19/83) for community acquired, 19% (11/57) for health care associated and 33% (19/58) for hospital acquired infections.

**Conclusion:**

Invasive vascular devices were associated with a substantial portion of cases of *S*. *aureus* bacteremia and provide an important target for infection control efforts.

## Introduction

*Staphylococcus aureus* colonizes 30% of the human population and frequently causes invasive infections, particularly in health care settings [1]. In the United States and Europe, the most commonly identified sources of *S*. *aureus* bacteremia (SAB) are vascular catheter-related infections, skin and soft tissue (SST) infections, pleuropulmonary infections, osteoarticular infections and infective endocarditis. However, a focus of infection is not found in ~25% of cases [2].

Risk factors, etiologies and foci of infection for community acquired SAB are substantially different from that of hospital acquired SAB in studies reported from North America and Europe, but limited information is available from sub-Saharan Africa [2, 3]. A previous report from AKUHN showed that *S. aureus* was the second most common cause of community onset bacteremia (after *Escherichia coli*), but was relatively infrequent as a cause of hospital acquired bacteremia (6% of bloodstream infections) [4]. However, that was done with an informatic approach using time from admission to the collection of the blood culture and was unable to distinguish between community acquired and community onset health care associated SAB.

SAB is notable for adverse outcomes and the potential for relapse after treatment. Many studies have shown that infectious disease consults [2], appropriate antimicrobial treatment and adherence to quality-of-care process measures are important in managing SAB [5]. These approaches should be guided by local information; yet, most studies of SAB have been performed in the United States and Europe and some of the features described in those regions may be different from sub-Saharan Africa. Sub-Saharan African countries often lack resources for proper diagnosis and treatment of many conditions, including SAB [6]. However, AKUHN is uniquely qualified for this study as it is an internationally accredited hospital and lab results are reported in an electronic system, making accurate data extraction possible [7]. This study provides an opportunity to better understand how SAB impacts a broad spectrum of patients in the region. Determining risk factors for acquiring SAB in the hospital will help identify at-risk patients and reduce bacteremia due to preventable causes. Early and appropriate treatment could help reduce complications and mortality, shorten hospital admission durations, reduce recurrent admissions and decrease unnecessary expenses. Obtaining information on current treatment practices and resistance patterns will guide treatment of SAB moving forward.

## Materials and methods

### The study site

Aga Khan University Hospital is an East African regional referral center with approximately 280 inpatient beds located in Nairobi, Kenya. It began as a private hospital and in 2004, post-graduate medical education was started. Currently, the medical staff is a mixture of private physicians and university faculty. The hospital offers a full range of services including 55 critical care beds of which 26 are intensive care level and the remainder are high dependency beds that provide an intermediate level of care between intensive care unit and the wards. Adult, pediatric and maternal services are included as well as inpatient and outpatient dialysis. The majority of patient come from Nairobi, but some come from throughout East Africa. The hospital was accredited by the Joint Commission International in 2013.

### Microbiology laboratory procedures

The laboratory has been internationally accredited by SANAS (South African National Accreditation System) since 2011 [7]. CLSI standards are used for susceptibility testing.

Blood cultures were incubated in a Bactec system (Becton Dickinson, Franklin Lakes, NJ, USA) that offers continuous monitoring of the cultures. After coagulase testing, positive cultures were placed onto the VitekII system (Biomeriux, Marcy-l'Étoile, France), for automated biochemical determination of species and for performing susceptibilities. Both cefoxitin and oxacillin susceptibilities were performed to confirm the presence of oxacillin resistance. Discrepant results for oxacillin and cefoxitin were reported as oxacillin resistant. Penicillin susceptibility was determined by beta-lactamase testing on the VitekII and supplemented by manually performed beta-lactamase testing. Clindamycin susceptibilities were confirmed by testing for inducible resistance on the VitekII with supplementation as required by disk diffusion. Disk diffusion testing was performed as needed to confirm any ambiguous susceptibilty results.

### Identification and categorization of patients with SAB

We performed a retrospective study of patients with a positive blood culture for *S*. *aureus* from September 2010 to December 2018. During this time period, all laboratory data were available on the electronic medical record. The written medical records were reviewed for demographics, underlying medical conditions, and hospital outcomes, and focus of infection. The written records did not always have complete information on pre-existing conditions and some patients had insufficient workup to determine the focus of infection; these limitations are acknowledged. The electronic records were reviewed to determine the antimicrobial agents and method of administration. For patients with more than one culture growing *S. aureus*, the first episode was chosen for analysis.

### Division into community onset (includes community acquired and healthcare associated) and hospital acquired

**Fig 1:**
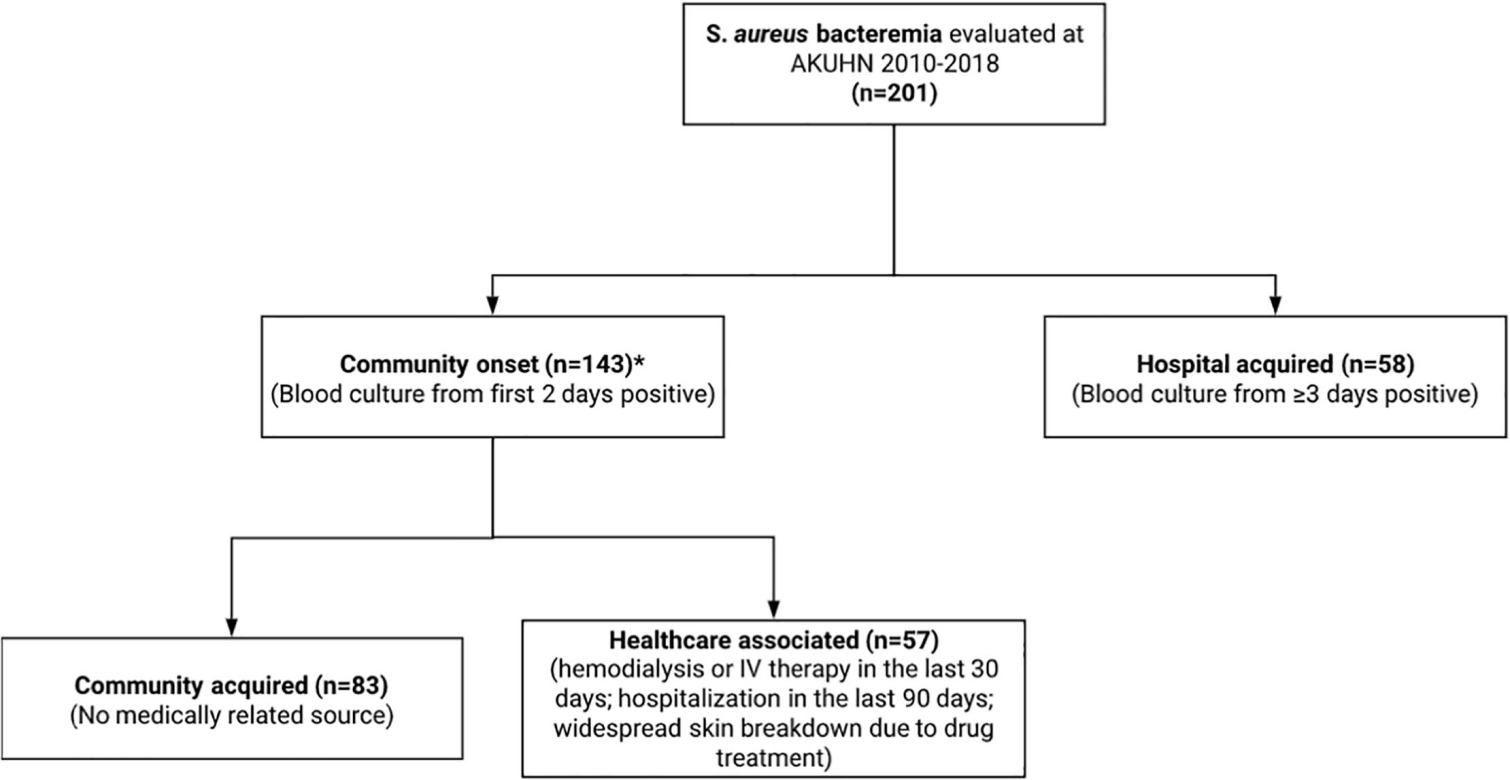
This figure describes the classification system used for SAB and the numbers of patients in each category.

Patients whose blood cultures grew *S. aureus* on admission or within the first two days were considered to have community onset SAB and were then assigned to either the community acquired (CA) or health care associated (HCA) categories (Fig 1). Those with no history of hospitalizations or medical interventions that might have provided a risk for SAB were considered to have CA SAB. Patients with early onset neonatal sepsis were included in the CA group. Most of the criteria proposed for defining HCA infections in [8, 9] were used, including intravenous therapy, wound care, chemotherapy within the last 30 days, hemodialysis or long term nursing home care. Hospitalization or nursing home stays within the last 90 days and transfers from other hospitals were considered HCA in this study. Table 1 compares the criteria used for healthcare associated *S. aureus* bacteremia in our study to current literature.

**Table 1:** Comparison of criteria used for healthcare associated *S. aureus* bacteremia.

Primary role of criteria	Cardoso [9]	Friedman et al [8]	Inclusion in current study
Risk for device-related infections	Received invasive procedures in the 30 days before the infection, including specialized nursing care	Received intravenous therapy at home; received wound care or specialized nursing care through a health care agency, family, or friends; or had self-administered intravenous medical therapy in the 30 days before the bloodstream infection. Patients whose only home therapy was oxygen use were excluded.	No examples in current study
Risk for device-related infections and colonization with resistant organisms	Attended a hospital or hemodialysis clinic in the previous 30 days;	Attended a hospital or hemodialysis clinic or received intravenous chemotherapy in the 30 days before the bloodstream infection.	These criteria were included
Colonization with resistant organisms	Were hospitalized in an acute care hospital for 2 or more days in the previous year;	Was hospitalized in an acute care hospital for 2 or more days in the 90 days before the bloodstream infection.	Yes, the 90-day cutoff is more applicable in the absence of evaluating for prolonged carriage of resistant organisms
Colonization with resistant organisms	Resided in a nursing home or long-term care facility;	Resided in a nursing home or long-term care facility.	No examples in current study (nursing homes are rare in this setting)
Colonization with resistant organisms	Treatment with broad spectrum antibiotics in the last 30 days.		This was not included since the information was usually missing and because this criterion is relevant for resistant organisms rather than SAB
Systemic infection due to skin breakdown			Widespread skin breakdown secondary to drug treatment. This is supported by the significant number of patients with SAB associated with skin breakdown

Patients with blood cultures obtained three or more days after admission to AKUHN were considered to have hospital acquired (HA) bacteremia unless there was evidence to the contrary from the medical record. The approach of including blood cultures collected after three days rather than the criterion of symptom onset within 48 hours has an accuracy of about 98% in comparison with the standard definition that includes onset of symptoms more than 48 hours after admission [10]. For patients with hospital-acquired bacteremia, standard National Healthcare Safety Network criteria were used to attribute a central venous catheter (CVC) as the source. For patients with peripheral intravenous catheters in place and no other likely source, the SAB was attributed to the catheter.

Treatment records were obtained from electronic and written records. However, the electronic records usually did not include discharge medications. Outpatient intravenous antibiotics were rarely available, so any discharge medications were oral.

### Ethical considerations

The protocol was reviewed and approved by the research ethics committee for AKUHN and was given the status as exempt from individual consent as a retrospective study of pre-existing data. All data have been de-identified for patient confidentiality. The research committee of AKUHN is accredited by the national accrediting body, National Council of Science and Technology (**NACOSTI**).

## Results

### Patient demographics

**Fig 2:**
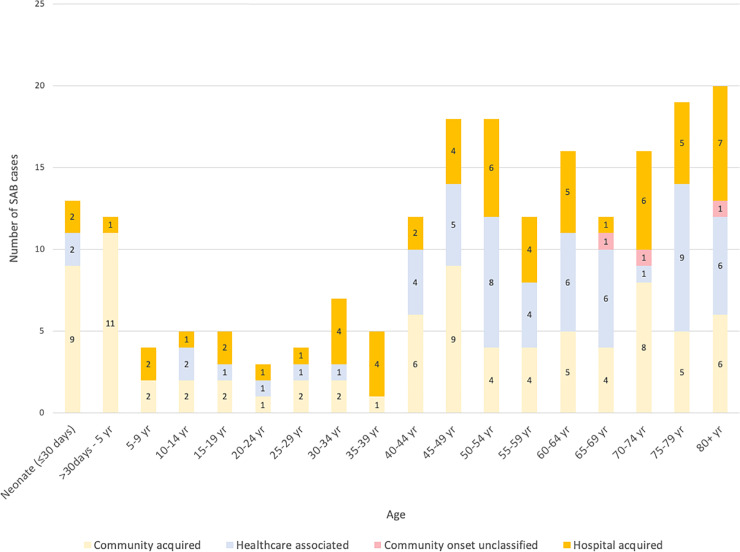
SAB by age group and type of infection. The number of cases by age are given in 5-year increments, except that age 0 to 4 is divided into two sets (<30 days and 31 days to 4 years), and all ages above 80 combined.

There were 201 patients with blood cultures positive for *S*. *aureus* who received care at AKUHN from September 2010 to December 2018. For the patients with more than one episode of SAB, data on etiology and focus of infection are reported for only the first episode. The number of patients per year (2011–2018) ranged from 17 to 34 with no clear trend from year to year. The most common comorbidities found in patients with SAB were hypertension (36%), diabetes (26%) and end stage renal failure (20%) (Table 2). Patient ages (ranging from birth to 103 years) were grouped into 5-year increments except that those aged 80 and above were combined into one group. When examined in these five-year segments, SAB was the most frequent in patients less than five years of age (n = 25, including 13 neonates); all but three cases were community acquired and were in patients without pre-existing conditions (Fig 2). The incidence of SAB was lower in patients 5 to 39 years of age, and incidence began to increase for patients 40 and over. The age group with the highest number of SAB cases was 80 years of age and over (20 cases). Among the patients over 45, hospital-acquired and healthcare associated predominated.

**Table 2:** Pre-existing conditions of patients with SAB.

		Community onset (n = 143)*		
**Condition n(%)**	Overall (n = 201)	Community acquired (n = 83)	Healthcare associated (n = 57)	Hospital acquired (n = 58)
Diabetes	53 (26%)	21 (25%)	20 (35%)	12 (21%)
Hypertension	72 (36%)	24 (29%)	30 (53%)	18 (31%)
HIV^1^	13 (6%)	3 (4%)	5 (9%)	5 (9%)
Surgery	28 (14%)	8 (10%)	16 (28%)	4 (7%)
Malignancy	14 (7%)	2 (2%)	7 (12%)	5 (9%)
End stage renal failure	40 (20%)	2 (2%)	28 (49%)	10 (17%)
Liver failure	3 (1%)	1 (1%)	0 (0%)	2 (3%)

Pre-existing conditions that were documented in the medical record

*Of the 143 community onset cases, 3 were not classified into healthcare associated or community acquired due to inadequate documentation in the medical record.

HTN, hypertension

HIV, human immunodeficiency virus

^1^Includes patients known to be HIV positive. Systematic HIV screening/testing was not conducted.

### Community acquired SAB

Of the 201 patients with SAB, 71% (143/201) were community onset. Of the 143 community onset cases, 58% were community acquired, 40% were healthcare assocaited, and 2% were not classified into either group due to inadequate documentation. CA SAB comprised the largest group of patients with SAB (41%; 83/201). The most commonly identified foci of infection for CA SAB cases were musculoskeletal (25%; 21/83) and skin and soft tissue (24%; 20/83), with smaller numbers of patients with central nervous system (CNS) or pulmonary presentations (Table 3). It is noteworthy that the sources for a large number (35%; 29/83) remained unknown.

**Table 3:** Site/Source of infection.

		Community onset (n = 143)*		
**Site/source of infection n(%)**	Overall (n = 201)	Community acquired (n = 83)	Healthcare associated (n = 57)	Hospital acquired (n = 58)
CNS	6 (3%)	6 (7%)	0 (0%)	0 (0%)
Endocarditis	3 (1%)	2 (2%)	1 (2%)	0 (0%)
Mediastinitis	1 (0%)	0 (0%)	0 (0%)	1 (2%)
Musculoskeletal	28 (14%)	21 (25%)	5 (9%)	2 (3%)
Pulmonary	5 (2%)	5 (6%)	0 (0%)	0 (0%)
SST	29 (14%)	20 (24%)	5 (9%)	4 (7%)
Unknown	46 (23%)	29 (35%)	6 (11%)	8 (14%)
Vascular	83 (41%)	0 (0%)	40 (70%)	43 (74%)

*Of the 143 community onset cases, 3 were not classified into healthcare associated or community acquired due to inadequate documentation in the medical record.

CNS, central nervous system

SST, skin and soft tissue

### Healthcare associated SAB

Vascular catheters constituted the majority of SAB that was HCA, and in this case, over half (53%; 30/57 of all HCA SAB) were associated with dialysis catheters (S1 Table). The relatively low portion due to other intravascular catheters may reflect the uncommon use of central venous catheters for outpatient cancer chemotherapy.

### Hospital acquired SAB

The standard definition of hospital acquired infection includes onset of symptoms at least 48 hours after hospitalization, but we used the correlate of blood culture positivity at three days or more [10]. We analyzed the records for SAB with onset at three days of hospitalization and found only one case of SAB with the first blood culture collected at three days. That patient had clinical evidence of SAB secondary to peripheral venous catheter, supporting the accuracy of this approach. Vascular catheters also comprised the majority (74%; 43/58) of HA SAB; of this group, 65% (28/43) of the infections were associated with peripheral catheters, while 33% (14/43) were associated with central catheters (routine CVC and/or dialysis catheters) and one was associated with a pacemaker (S1 Table).

### Foci and sources of SAB

#### SAB due to skin disease

Nineteen of the 22 patients (86%) with SAB related to skin disease had onset of bacteremia prior to admission to AKUHN, but four of these were related to prior medical therapy and were considered health care associated (see S2 Table for brief descriptions of some of the patients).

#### Bone and joint infections

All 11 (eight with CA and three with HCA SAB) patients in this study with osteomyelitis-associated SAB had community onset infection and had just a single focus of infection, including humerus (2), vertebral or paravertebral (2), femur, heel, knee, ankle, toe and skull. Nine patients had joint-related SAB; two of these were total joint arthroplasties. Of the seven cases associated with native joint infections, six were CA while one was HA (S1 Table).

### Susceptibility

Susceptibility data were available for all 201 isolates. Ninety percent of isolates were oxacillin-susceptible *S*. *aureus* (OSSA) while 12% were susceptible to penicillin G (Table 4). The isolates were uniformly susceptible to vancomycin and linezolid and 85% were susceptible to clindamycin, including 53% of the oxacillin-resistant *S*. *aureus* (ORSA).

**Table 4:** Susceptibilities.

			Community onset (n = 143)*					
	Overall		Community acquired (n = 83)		Healthcare associated (n = 57)		Hospital acquired (n = 58)	
** Drug**	n	% Susceptible	n	% Susceptible	n	% Susceptible	n	% Susceptible
Linezolid	200	100%	83	100%	57	100%	57	100%
Vancomycin	201	100%	83	100%	57	100%	58	100%
Quinolone	162	92%	66	92%	46	89%	47	94%
Oxacillin	196	90%	79	94%	56	86%	58	90%
Tetracycline	181	87%	73	86%	52	87%	53	87%
Clindamycin^1^	200	85%	83	90%	56	82%	58	79%
Erythromycin	201	82%	83	87%	57	77%	58	79%
TMP/SMX	157	64%	62	65%	45	64%	49	63%
Penicillin	197	12%	80	10%	56	18%	58	9%

^1^53% (10/19) of the ORSA isolates were susceptible to clindamycin

*Of the 143 community onset cases, 3 were not classified into healthcare associated or community acquired due to inadequate documentation in the medical record.

### Treatment and outcome

The overall in-hospital mortality was 25% (50/201) and ranged from 19% (11/57) for HCA to 33% (19/58) for HA, which was not a statistically significant difference (Table 5). Mortality was also similar for patients with ORSA (26%, 5/19) or OSSA (25%, 45/177) bacteremia.

**Table 5:** Outcome for SAB.

		Community onset*		
	Overall	Community Acquired	Health Care Associated	Hospital Acquired
**Died**	50	19	11	19
**Discharged**	143	62	42	37
**Not admitted**	3	1	2	0
**Transferred**	5	1	2	2
**Total**	201	83	57	58
**Mortality Rate**	25%	23%	19%	33%

p = 0.14 for mortality of HCA vs. HA, 0.68 for HCA vs. CA, 0.25 for HA vs. CA; Fisher’s exact two-tailed test

HCA, healthcare associated

HA, hospital acquired

CA, community acquired

*Of the 143 community onset cases, 3 were not classified into healthcare associated or community acquired due to inadequate documentation in the medical record.

**Fig 3:**
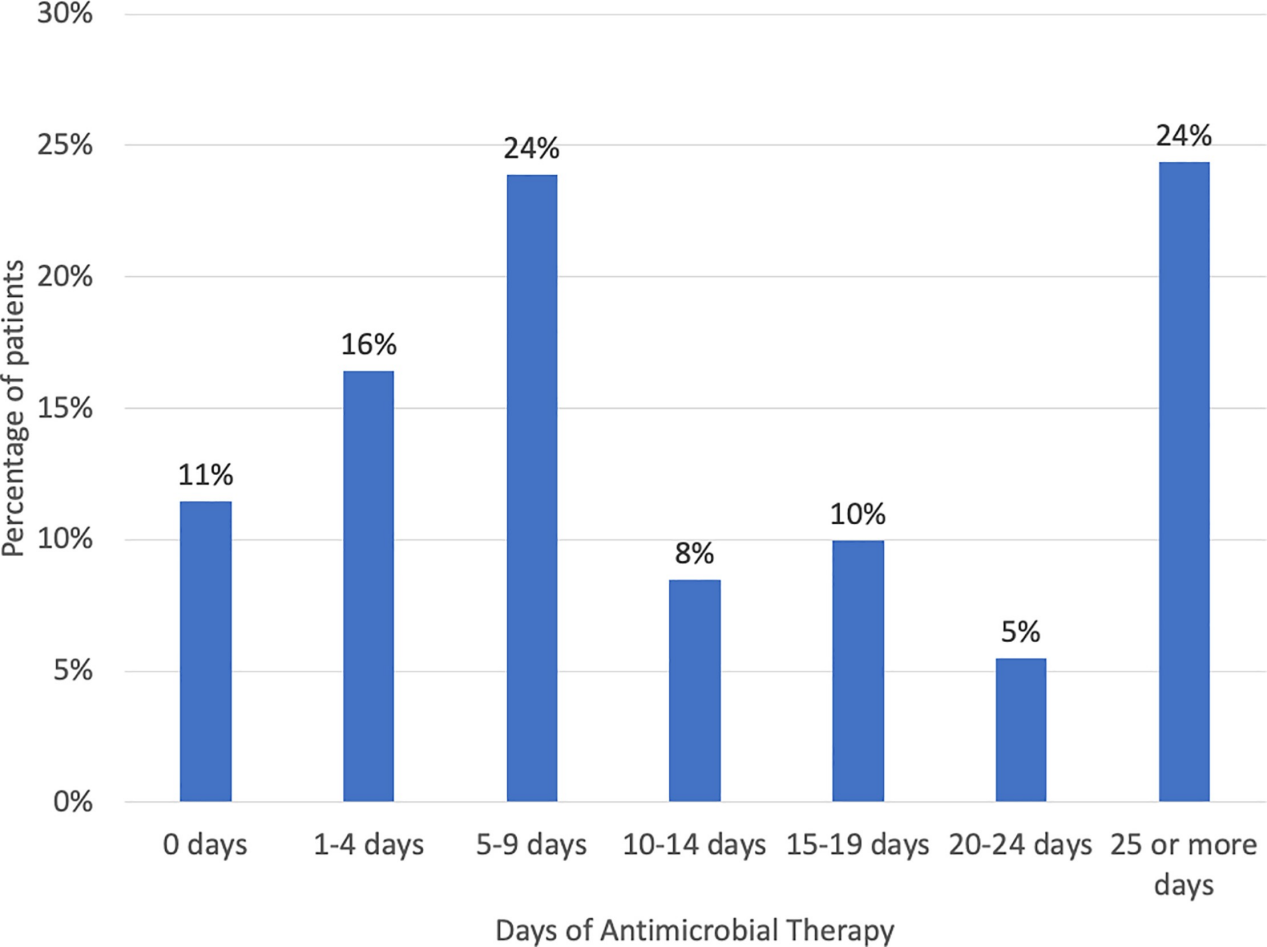
Duration of treatment for SAB. Drugs with demonstrated efficacy for staphyloccoccal infections, including anti-staphylococcal penicillins, penicillins with beta-lactamase inhibitors, cephalosporins, carbapenems, glycopeptides and linezolid were included. Both oral and intravenous formulations were included.

The typical recommendations are to treat for two weeks (or 10–14 days) for uncomplicated SAB and for at least four weeks for complicated SAB, including endocarditis [2]. However, in this analysis, treatment days with an agent that has demonstrated efficacy for staphylococcal infections were included, including oral therapy (eg. oral flucloxacillin, amoxicillin/clavulanate, clindamycin, etc). Despite this liberal definition of days of therapy, only 48% (97/201) were treated for 10 days or longer (Fig 3). The three patients who did not receive antimicrobial treatment died on the same day the positive culture was collected. Of the five patients with ORSA bacteremia who were admitted to the hospital and died, two died too early for directed therapy, and the other three were treated with a glycopeptide.

## Discussion

*S. aureus* is one of the most common etiologies of both community onset and hospital acquired bloodstream infections in most series reported in the literature. However, evidence from our own institution [4] and elsewhere in Kenya [11] suggest that *S*. *aureus* is less prominent in sub-Saharan Africa than in the West. An epidemiologic study of bacteremia in children under 13 years of age in Kenya found that *S. aureus* was the fifth most common cause [11]. In a series from our institution, *S. aureus* ranked second as a cause of community onset bloodstream infection, but only fifth among hospital acquired organisms [4]. Worldwide, *S*. *aureus* is a major cause of mortality related to bacteremia, with reported mortality rates in the range of 20–30% [12], although there is wide variation among studies related to the types of staphylococcal infections seen. Relatively little is known about SAB from sub-Saharan Africa outside South Africa. Therefore, we performed this retrospective review to determine the etiologies and clinical course for SAB.

Throughout many of the high-resource countries, ORSA comprises a substantial percentage of *S. aureus* bacteremia cases. For example, an analysis of S. *aureus* bacteremia from 124 Veterans Administration hospitals in the United States showed that 52% were caused by ORSA. In South African children with SAB, ORSA was common (39%) in community acquired SAB bacteremia, and HIV infection was identified as a risk factor [13]. In another South African study of SAB that included all ages and included hospital acquired SAB, ORSA was also common (36%) and HIV infection was a prominent risk factor [14]. The low prevalence of ORSA (10%) among the SAB cases in the current series differs markedly from Europe, North America and South Africa. In fact, the current series had more cases that were penicillin susceptible (12%) than ORSA (10%). The ORSA rate at AKUHN for all isolates is similar to that of blood isolates and has shown an increase over time from 4% in 2012 to 12% in 2017 [4] and (R. Adam, unpublished data).

The low percentage of ORSA is consistent with the low rate of ORSA colonization [15] and the low ORSA rates seen in a city near Nairobi [16]. It is also consistent with the low prevalence of ORSA (0/14) in a study of bacteremia in The Gambia reported in 2007 [17]. However, it does contrast with a recent report of a rate of 53% ORSA in a study of the susceptibility of *S*. *aureus* from a national referral hospital in Nairobi [18]. The reasons for the differences are not clear.

Whether HIV infection is a risk factor for the population in the current study is not clear in that the HIV prevalence rate in our study (6%) is only slightly higher than the adult (ages 15–49) HIV prevelance rate in Kenya of 4.7% [19] However, meaningful comparison of these rates is limited given the more expansive age range, small sample size, and lack of routine HIV testing in our cohort.

Most cases of SAB in the current study were community onset cases (71%; 143/201), but many of those cases were health care associated (28% of the total; 57/201). For these HCA cases, over half (53%; 30/57) were associated with dialysis catheters. The prominence of dialysis catheters as sources of SAB is particularly noteworthy in view of the growing burden of noncommunicable diseases in sub-Saharan Africa and the plan of the Kenyan government to expand access to dialysis across the country [20, 21]. This is a potential source of morbidity that must be monitored. In fact, the large number of patients in the current series with hypertension (36%; 72/201), diabetes (26%; 53/201) or end stage renal failure (20%; 40/201) points to this growing epidemic. Of the community acquired cases, nearly half (49%; 41/83) had foci/sources that were skin and soft tissue or musculoskeletal.

The rarity of *S*. *aureus* endocarditis (only three patients) in the current case series contrasts with series reported from elsewhere [22, 23]. Of the HA cases, 48% (28/58) were associated with peripheral intravenous catheters. For this study, we considered the peripheral intravenous catheter as the source when the patient had a peripheral line and no other likely source of infection, similar to the approach in a previous study of peripheral catheters and SAB [24]. While this approach may overestimate the role of the peripheral intravenous catheter, an approach requiring the presence of phlebitis is likely to underestimate the frequency, especially since erythema is often missed in dark-skinned patients [25]. In contrast to the peripheral intravenous lines, *Candida* species and Gram-negative organisms are much more common as causes of central line-associated bloodstream infections at AKUHN [26] and (R. Adam, unpublished data).

The short duration of treatment given many patients in the current series, with only 48% (97/201) of patients receiving the generally accepted minimum treatment duration of 10 days or longer, contrasts with recommendations from the literature. The typical recommendations are to treat for two weeks (or 10–14 days) for uncomplicated SAB and for at least four weeks for complicated SAB, including endocarditis [27]. The preferred treatment regimen includes an intravenous antistaphylococcal penicillin or first-generation cephalosporin for OSSA and vancomycin for ORSA, although other treatment choices are sometimes advocated [27]. By these criteria, a relatively small portion of the patients received what would be considered optimal therapy. These data from sub-Saharan Africa point out the important role of health care treatment in the development of SAB, not only in the obvious dialysis-associated SAB, but also those cases resulting from extensive skin breakdown from drug reactions. Vigilant infection control practices will be required to prevent escalation of this problem in the future.

A major strength of this study is that the internationally accredited laboratory and availability of electronic records of microbiology results mean that accurate diagnosis and susceptibility was available. As the only study to date of patients hospitalized with *S*. *aureus* bacteremia in sub-Saharan Africa, it provides an important perspective on differences from series reported in high resource countries. The major weaknesses include: (1) As a private hospital, the spectrum of *S*. *aureus* disease may differ from what is seen in other facilities, (2) Because of the retrospective nature of the study, prior medical treatment and risk factors were often unavailable, treatment records may underestimate the actual treatment given, and post-hospitalization outcome was not routinely available.

## Conclusions

This study points out the important role of health care-related complications as risk factors for SAB, especially related to hemodialysis. With the expansion of hemodialysis to regions throughout Kenya, infection control practices will need to address the risks of SAB in these settings.

## Supporting information

S1 Table Detailed list of sources/sites. (DOCX)

S2 Table Description of patients with skin conditions associated with SAB. (DOCX)

S1 Data (CSV)

## Abbreviations used

AKUHN: (Aga Khan University Hospital Nairobi
CA: (community acquired)
CNS: (central nervous system)
CVC: (central venous catheter)
HA: (hospital acquired)
HCA: (Health care associated)
HIV: (human immunodeficiency virus)
ORSA: (oxacillin-resistant *S*. *aureus*)
OSSA: (oxacillin-susceptible *S*. *aureus*)
SAB: (*Staphylococcus aureus* bacteremia)
SJS: (Stevens Johnson Syndrome)
SST: (skin and soft tissue)
