# Supplementary material for: Staphylococcus aureus bacteremia at a referral medical center in Kenya: A retrospective review of cases from 2010 to 2018
Source: PLoS One. 2020 Jun 23;15(6):e0234914. doi: 10.1371/journal.pone.0234914 (PMC7310726; doi:10.1371/journal.pone.0234914)
Supplement: S1 Table — (DOCX) [file pone.0234914.s001.docx]

**Supplemental Table 1: Detailed list of sources/sites**

|  |  | **Community onset*** | |  |  |
| --- | --- | --- | --- | --- | --- |
| **Abbreviated site/source** | **Detailed Site/Source** | **Community acquired** | **Healthcare associated** | **Hospital acquired** | **Total** |
| CNS | Epidural empyema | 2 | 0 | 0 | 2 |
| CNS | Meningitis | 2 | 0 | 0 | 2 |
| CNS | Orbital cellulitis | 2 | 0 | 0 | 2 |
| Endocarditis | Native valve endocarditis | 2 | 0 | 0 | 2 |
| Endocarditis | Prosthetic valve endocarditis | 0 | 1 | 0 | 1 |
| Mediastinitis | Mediastinitis | 0 | 0 | 1 | 1 |
| Musculoskeletal | Muscle | 7 | 0 | 1 | 8 |
| Musculoskeletal | Osteomyelitis | 8 | 3 | 0 | 11 |
| Musculoskeletal | Septic native joint | 6 | 0 | 1 | 7 |
| Musculoskeletal | Total joint arthroplasty | 0 | 2 | 0 | 2 |
| Pulmonary | Pulmonary | 5 | 0 | 0 | 5 |
| SST | Skin | 15 | 4 | 3 | 22 |
| SST | Subcutaneous | 3 | 0 | 0 | 3 |
| SST | Wound | 2 | 1 | 1 | 4 |
| Unknown | Unknown | 29 | 6 | 8 | 46 |
| Vascular | CVC | 0 | 5 | 11 | 16 |
| Vascular | Dialysis catheter | 0 | 30 | 3 | 33 |
| Vascular | Pacemaker | 0 | 5 | 1 | 6 |
| Vascular | Peripheral IV | 0 | 0 | 28 | 28 |
| Grand Total |  | 83 | 57 | 58 | 201 |

*Of the 143 community onset cases, 3 were not classified into healthcare associated or community acquired due to inadequate documentation in the medical record.

CNS, central nervous system

SST, skin and soft tissue

CVC, central venous catheter

IV, intravenous catheter
