# Supplementary material for: Staphylococcus aureus bacteremia at a referral medical center in Kenya: A retrospective review of cases from 2010 to 2018
Source: PLoS One. 2020 Jun 23;15(6):e0234914. doi: 10.1371/journal.pone.0234914 (PMC7310726; doi:10.1371/journal.pone.0234914)
Supplement: S2 Table — (DOCX) [file pone.0234914.s002.docx]

**Supplemental Table 2: Description of skin conditions associated with SAB**

| **Type** | **Description** |
| --- | --- |
| CA | A neonate with a diffuse, pustular skin rash |
| CA | An infant with bullous skin lesions |
| CA | A child with a fever and rash for two weeks and a diagnosis of impetigo |
| HA | An adult male who developed Stevens Johnson syndrome after receiving amoxicillin/clavulanate |
| HA | SJS with CVC-related bacteremia |
| HA | SJS associated with TMP/SMX prophylaxis in HIV |
| HA | Burn wounds; bacteremia related to peripheral IV |
| HA | A female with discoid lupus erythematosus (DLE) and mixed connective tissue disease and MRSA infection of skin wounds attributed to her underlying skin disease |
| HCA | A child with full-thickness burns covering 25% of the body |
| HCA | An adult male with SJS possibly due to a sulfa drug |
| HCA | An adult female with ascending myelitis who developed desquamating skin lesions after receiving phenytoin |
| HCA | An adult with toxic epidermal necrosis |
| HCA | A female HIV patient with facial puffiness and blistering and jaundice |

CA, community acquired

CVC, central venous catheter

HA, hospital acquired

HCA, healthcare associated

HIV, human immunodeficiency virus

IV, intravenous catheter

MRSA, methicillin-resistant S. *aureus*

SJS, Stevens Johnson Syndrome

TMP/SMX, trimethoprim-sulfamethoxazole
